# MiR-130a-3p regulates cell migration and invasion via inhibition of Smad4 in gemcitabine resistant hepatoma cells

**DOI:** 10.1186/s13046-016-0296-0

**Published:** 2016-01-27

**Authors:** Yang Liu, Yumei Li, Rui Wang, Shukui Qin, Jing Liu, Fang Su, Yan Yang, Fuyou Zhao, Zishu Wang, Qiong Wu

**Affiliations:** Department of Medical Oncology, First Affiliated Hospital of Bengbu Medical College, Bengbu, Anhui China; Department of Medical Oncology, PLA Cancer Center, Nanjing Bayi Hospital, Nanjing, Jiangsu China

**Keywords:** Hepatocellular carcinoma, EMT, Smad4, miR-130a-3p, Invasion

## Abstract

**Background:**

Emerging evidence demonstrates that microRNAs (miRNAs) play an important role in regulation of cell growth, invasion and metastasis through inhibiting the expression of their targets. It has been reported that miR-130a-3p controls cell growth, migration and invasion in a variety of cancer cells. However, it is unclear whether miR-130a-3p regulates epithelial-mesenchymal transition (EMT) in drug resistant cancer cells. Therefore, in the current study, we explore the role and molecular mechanisms of miR-130a-3p in gemcitabine resistant (GR) hepatocellular carcinoma (HCC) cells.

**Methods:**

The real-time RT-PCR was used to measure the miR-130a-3p expression in GR HCC cells compared with their parental cells. The wound healing assay was conducted to determine the cell migratory activity in GR HCC cells treated with miR-130a-3p mimics. The migration and invasion assays were also performed to explore the role of miR-130a-3p in GR HCC cells. Western blotting analysis was used to measure the expression of Smad4, E-cadherin, Vimentin, and MMP-2 in GR HCC cells after depletion of Smad4. The luciferase assay was conducted to validate whether Smad4 is a target of miR-130a-3p. The student *t*-test was used to analyze our data.

**Results:**

We found the down-regulation of miR-130a-3p in GR HCC cells. Moreover, we validate the Smad4 as a potential target of miR-130a-3p. Furthermore, overexpression of miR-130a-3p suppressed Smad4 expression, whereas inhibition of miR-130a-3p increased Smad4 expression. Consistently, overexpression of miR-130a-3p or down-regulation of Smad4 suppressed the cell detachment, attachment, migration, and invasion in GR HCC cells.

**Conclusions:**

Our findings provide a molecular insight on understanding drug resistance in HCC cells. Therefore, activation of miR-130a-3p or inactivation of Smad4 could be a novel approach for the treatment of HCC.

## Background

Liver cancer is one of the most frequently diagnosed cancers in the United States [1]. An estimated 35,660 new cases and 24,550 cancer deaths will be occurred from liver cancer in 2015 in the US [1]. It has been reported that 782,500 new liver cancer cases and 745,500 deaths occurred worldwide during 2012, and half of the total number of cases and deaths are from China [2]. Most primary liver cancers are hepatocellular carcinoma (HCC). The some factors have been reported to contribute to HCC, such as aflatoxin, chronic hepatitis B virus infection, obesity, type 2 diabetes, heavy alcohol consumption, and smoking [3, 4]. The treatments for HCC include surgical resection, liver transplantation and ablation, and chemotherapy. Since most HCC patients are diagnosed at the advanced stages, systemic chemotherapy is required. However, HCC patients often acquire drug resistance to chemotherapy, leading to limited survival benefits, suggesting that understanding and overcoming drug resistance is necessary to improve the treatment of HCC.

It has been known that miRNAs exert their regulatory functions through binding to the 3′ untranslated region (3′UTR) of target mRNA, leading to the degradation of the mRNA or translational inhibition of functional proteins. Emerging evidence has demonstrated that miRNAs are critically involved in the development and progression of human cancers including HCC [5–7]. For example, miR-93 promotes cell proliferation via targeting PTEN in osteosarcoma cells [5]. One study showed that miR-106b promotes colorectal cancer cell migration and invasion through directly targeting DLC1 [6]. Recently, a growing body of data implicates that micorRNAs (miRNAs) play an essential role in drug resistance through inhibition of its downstream targets in human cancers [8, 9]. For example, upregulated miR-182 increases drug resistance in cisplatin-treated HCC cells through regulation of TP53INP1 [10]. Furthermore, miR-340 reversed cisplatin resistance of HCC cell lines via regulating Nrf2-dependent antioxidant pathway [11]. Liu et al. found that miR-222 regulates sorafenib resistance and enhances tumorigenicity in HCC through governing PI3K/Akt pathway [12]. Another study revealed that restoration of miR-193b sensitizes HBV-associated HCC cells to sorafenib [13]. Moreover, reprogramming using miR-302 improves drug sensitivity in HCC cells through down-regulation of AOF2, which caused H3K4 methylation and c-Myc repression [14]. Yang et al. found that miR-223 modulated multidrug resistance by downregulation of ABCB1 in HCC cells [15]. Emerging evidence has also suggested that miR-338-3p [16], let-7b [17], miR-942 [18], miR-612 [19], miR-21 [20], miR-26b [21], miR-34a [22], miR-216a/217 [23], miR-193a-3p [24, 25], and miR-122 [26, 27] are critically involved in drug resistance in HCC cells. These reports define that targeting miRNAs could be a promising approach to overcome drug resistance.

Early studies reported that miR-130a was directly involved in the development of chemoresistance [28–30]. Down-regulation of miR-130a was found in a panel of paclitaxel- and cisplatin-resistant ovarian cancer cells [28, 31], gefitinib-resistant non-small cell lung cancer (NSCLC) cells [32], and paclitaxel-resistant prostate cancer cells [33]. Consistently, docetaxel-resistant head and neck squamous cell carcinoma cell lines have lower expression of miR-130a-3p compared with their parental cells [29]. Moreover, it has been reported that miR-130a targets MET and induces TRAIL-sensitivity in NSCLC cells [30]. Furthermore, one study showed that miR-130a was down-regulated in triple-negative breast cancer, which is associated with chemoresistance [34]. Interestingly, miR-130a was found to be upregulated in cisplatin-resistant ovarian cancer cells [35, 36]. Although these studies indicated miR-130a as a key role in chemoresistance in human cancer cells, the molecular mechanism of miR-130a-meidated drug resistance has not been fully elucidated. Therefore, the goal of this study was to explore the role of miR-130a-3p in gemcitabine resistant (GR) hepatocellular carcinoma (HCC) cells. We found the down-regulation of miR-130a-3p in GR HCC cells. More importantly, Smad4 was validated as a potential target of miR-130a-3p. Notably, overexpression of miR-130a-3p suppressed the cell migration, and invasion in GR HCC cells partly through down-regulation of Smad4. Our findings suggest that activation of miR-130a-3p or inactivation of Smad4 could be a novel approach for the treatment of HCC.

## Methods

### Cell culture, reagents and antibodies

HepG2, HepG2 GR, SMMC-7721, SMMC-7721 GR cells were cultured at 37 °C in 5 % CO_2_ in Dulbecco’s modified Eagle’s medium (DMEM; Gibco, Gaithersburg, MD, USA) supplemented with 10 % fetal bovine serum. The secondary antibodies and primary antibodies against Smad4, MMP2 and GAPDH were bought from Santa Cruz Biotechnology (Santa Cruz, CA). Anti-Vimentin, anti-E-cadherin antibodies were obtained from Abcam.

### Wound healing assay

The HCC cells and HCC GR cells were seeded in 6-well plate until the cells grew to 90–95 % confluency. The scratch wound was generated in the surface of the plates using a pipette tip in cells with miR-130a-3p mimic transfection or Smad4 siRNA treatment. Photographic images were taken from HCC and HCC GR cells at 0 and 20 h.

### Cell attachment and detachment assay

Cell attachment and detachment assays were conducted as described before [37–39]. Briefly, for attachment assay, HCC GR cells transfected with Smad4 siRNA or miR-130a-3p mimics were seeded in 24-well plates at 5 × 10^4^ cells per well. Unattached cells were removed after 1 h incubation, and the attached cells were counted after trypsinization. The data were presented as a percentage of the attached cells compared to total cells. For cell detachment assay, after 24 h incubation, the cells were incubated with 0.05 % trypsin for 3 min to detach the cells. Then, the culture medium was added to inactivate the trypsin and the detached cells were collected. The remaining cells were incubated with 0.25 % trypsin to detach and counted. The data were presented as a percentage of the detached cells to total cells.

### Transwell migration and invasion assays

The migration of HCC cells was conducted using a 24-well Transwell chamber (Corning) with gelatin-coated polycarbonate membrane filter. The invasive capacity of HCC cells was performed using Transwell inserts with Matrigel (BD Biosciences). After incubation for 20 h, the upper surfaces of the Transwell chambers were scraped with cotton swabs, and the migrated and invaded cells were fixed with 4 % paraformaldehyde, and then stained with Giemsa solution. The stained cells were photographed and counted under a light microscope in five randomly-selected fields.

### RNA extraction and reverse transcription-PCR analysis for gene expression

The total RNA from HCC cells and HCC GR cells was isolated with Trizol (Invitrogen) and purified with RNeasy Mini Kit and RNase-free DNase Set (Qiagen) according to the manufacturer’s protocols. The primer sequences for target genes were as follows: Smad4 (forward, 5′-CGG ACA TTA CTG GCC TGT TC-3′; reverse, 5′-TAG GGC AGC TTG AAG GAA ACC-3′; E-cadherin (forward, 5′-GAA GTG TCC GAG GAC TTT GG -3′; reverse, 5′-CAG TGT CTC TCC AAA TCC GAT A -3′); MMP-2 (forward, 5′-TAT GGC TTC TGC CCT GAG AC -3′; reverse, 5′-CAC ACC ACA TCT TTC CGT CA -3′); Vimentin (forward, 5′-TGT CCA AAT CGA TGT GGA TGT TTC-3′; reverse, 5′-TTG TAC CAT TCT TCT GCC TCC TG-3′); GAPDH (forward, 5′-CAG CCT CAA GAT CAT CAG CA -3′; reverse, 5′-TGT GGT CAT GAG TCC TTC CA-3′). The expression of GAPDH was used as internal control. RT-PCR amplifications were performed as described before [40].

### Protein extraction and Western blotting

Cells were harvested and lysed with RIPA buffer (1 × PBS, 1 % Nonidet P40, 0.5 % sodium deoxycholate, 0.1 % SDS, and protease inhibitor cocktail). The protein concentrations were measured using the Bio-Rad protein assay kit (Bio-Rad Laboratories, CA). Immunoblotting was conducted with standard protocols as described previously [41].

### miRNA real-time RT-PCR

The miRNA real-time RT-PCR assay was performed using miR-130a-3p TaqMan MicroRNA Assay Kit (Applied Biosystems). Briefly, ten nanogram of total RNA was reverse transcribed into cDNA and then real-time PCR was performed using specific primers for miR-130a-3p as described previously [37]. U6 was used as an endogenous control for normalization.

### Transfection

Cells were seeded in six-well plates and transfected with different Smad4 siRNAs (Genepharma, Shanghai, China), miR-130a-3p mimics and miR-130a-3p inhibitors (Genepharma, Shanghai, China) using Lipofectamine 2000 as described earlier [40]. After the indicated periods of incubation, the cells were subjected to further analysis as presented under the results section.

### Luciferase assays

The wild-type and mutant Smad4 3′-UTR were amplified by PCR and cloned in pMIR-REPORT (Ambion) with firefly luciferase. A total of 5 × 10^4^ cells treated with control, miR-130a-3p mimics, or miR-130a-3p inhibitors were transfected with wild-type or mutants of Smad4 3′ UTR luciferase reporters together with Renilla plasmid. After 48 h of transfection, the firefly and Renilla luciferase were measured according to the manufacturer’s protocols (Promega). The firefly luciferase activities were normalized to Renilla luciferase activities.

### Statistical analysis

All experiments were repeated at least 3 times. Values were shown as means ± SEM and analyzed using GraphPad Prism 4.0 (Graph pad Software, La Jolla, CA). Statistical comparisons between different groups were performed using Student *t* test. *P* < 0.05 was considered statistically significant.

## Results

### Down-regulation of miR-130a-3p in HCC GR cells

**Fig. 1:**
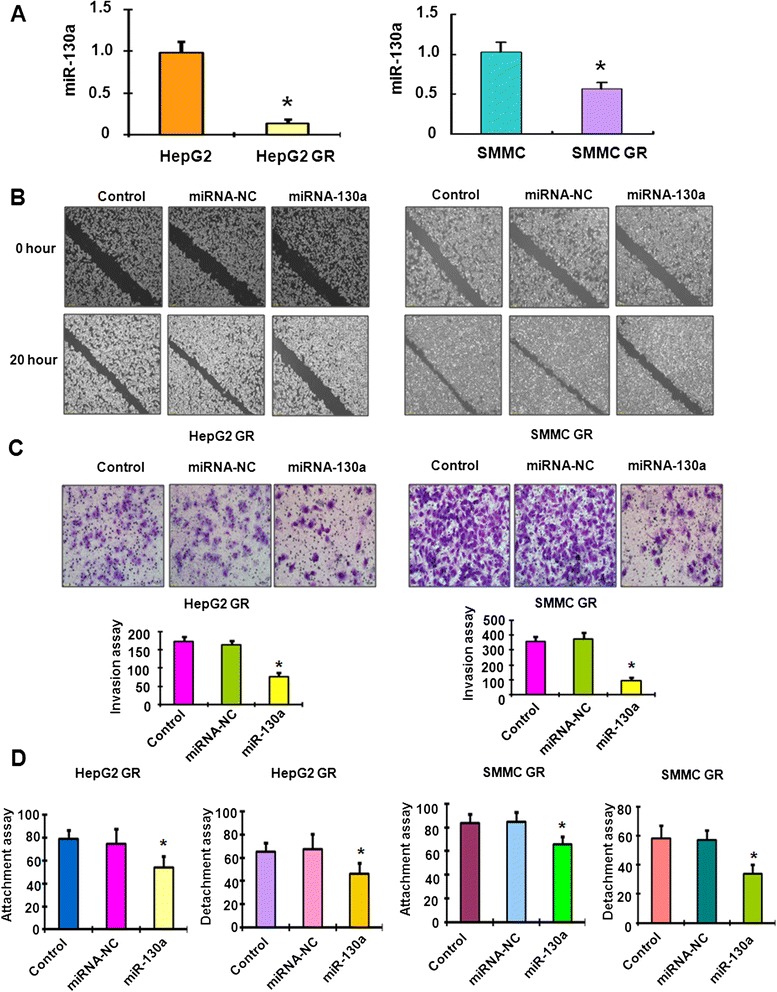
Down-regulation of miR-130a-3p in HCC GR cells. **a** Real-time RT-PCR assay was performed to detect the levels of miR-130a-3p in HCC and HCC GR cells. *p < 0.05, vs HCC cells. **b** Wound assays were performed to compare the migratory potential of HepG2 GR and SMMC-7721 GR cells after miR-130a-3p mimics treatment. **c** Top panel: Invasion assay was conducted to measure the invasive capacity in HepG2 GR and SMMC-7721 GR cells after miR-130a-3p mimics treatment. Bottom panel: Quantitative results are illustrated for top panel. *P < 0.05, vs control. **d** Cell attachment and detachment assays were conducted in HepG2 GR and SMMC-7721 GR cells after miR-130a-3p mimics treatment. *P < 0.05, vs control

First, the miRNA array in both HepG2 GR and HepG2 cells was performed. We found that multiple miRNAs were down-regulated and some miRNAs were up-regulate in HepG2 GR cells (data not shown). This finding indicates that further investigations are required to explore the mechanisms of GR-mediated miRNA dysregulation. Notably, miR-130a-3p expression was significantly down-regulated in HepG2 GR cells. It has been reported that miR-130a was critically involved in drug resistance [32, 33]. Therefore, we validated whether miR-130a-3p has changes in HCC GR cells compared with their parental cells. Our real-time RT-PCR results showed that miR-130a-3p was down-regulated in both HepG2 GR and SMMC-7721 GR cells (Fig. 1a). Recently, miR-130a was found to inhibit cell migration and invasion in human breast cancer cells [42]. In line with this finding, our wound-healing assay showed that miR-130a-3p mimics significantly decreased numbers of cells migrating across the wound in HepG2 GR and SMMC 7721 GR cells (Fig. 1b). Moreover, our invasion assay results revealed that miR-130a-3p mimics suppressed cell invasion in HCC GR cells compared with control miRNA treatment (Fig. 1c). Additionally, we observed that miR-130a-3p mimics inhibited the cell detachment and attachment in both HCC GR cells (Fig. 1d).

### Smad4 is negatively associated with miR-130a-3p expression

**Fig. 2:**
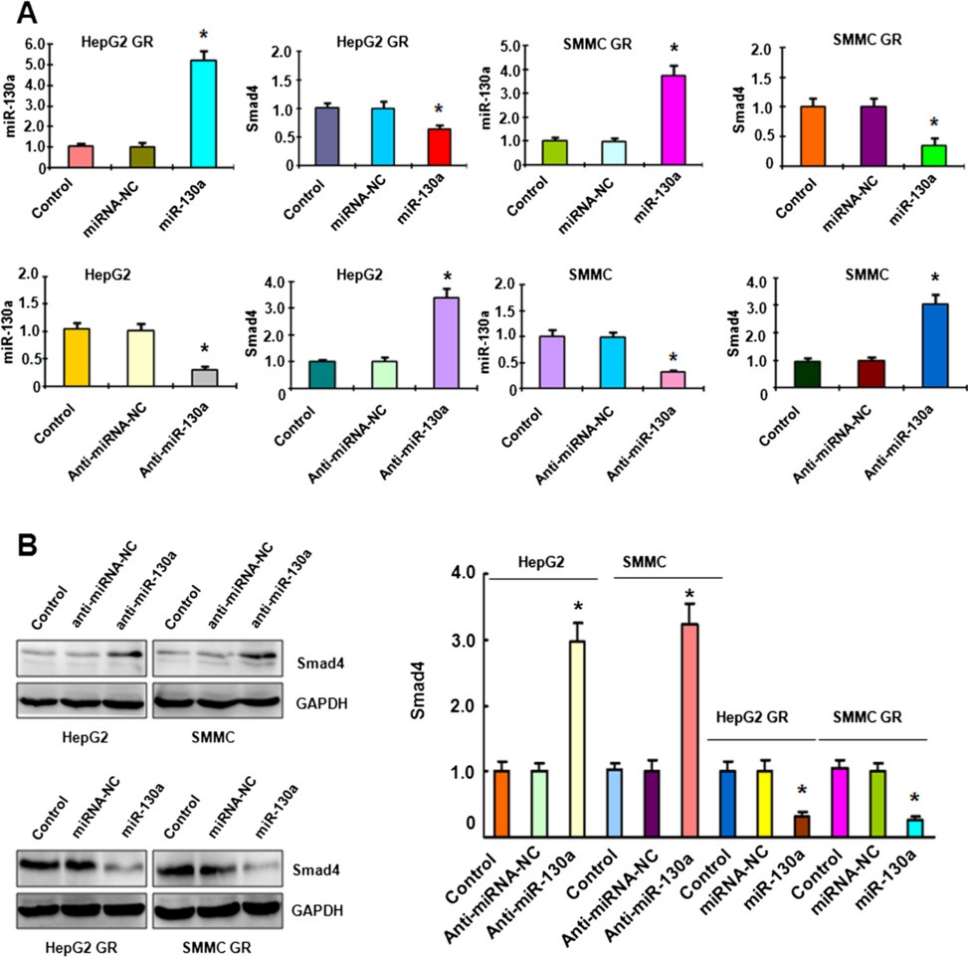
Smad4 is associated with miR-130a-3p expression. **a** Top panel: Real-time RT-PCR assay was performed to detect the mRNA level of Smad4 in HCC GR cells treated with miR-130a-3p mimics. miR-130a-3p was measured by miRNA real-time RT-PCR in HCC GR cells after miR-130a-3p mimic transfection. Bottom panel: Real-time RT-PCR assay was performed to detect the mRNA level of Smad4 in HCC cells treated with miR-130a-3p inhibitor. miR-130a-3p was measured by miRNA real-time RT-PCR in HCC cells after miR-130a-3p inhibitor treatment. *p < 0.05, vs control. **b** Left panel: Western blotting analysis was conducted to measure the expression of Smad4 in HCC cells treated with miR-130a-3p inhibitor and in HCC GR cells treated with miR-130a-3p mimic. Right panel: Quantitative results are illustrated for left panel

**Fig. 3:**
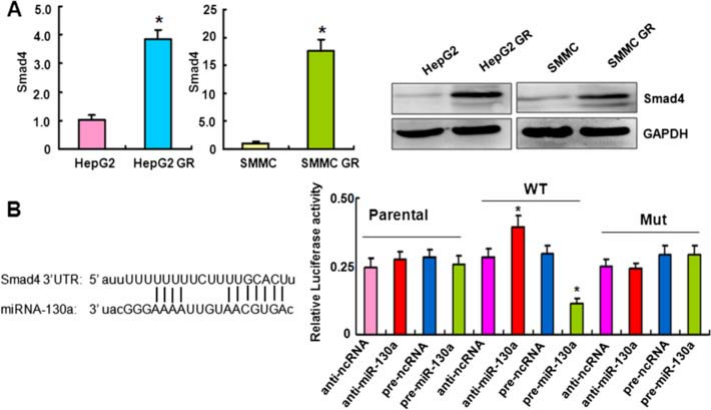
Smad4 is a downstream target of miR-130a-3p. **a** Left panel: Real-time RT-PCR assay was used to detect the mRNA level of Smad4 in HCC GR cells. *p < 0.05 vs control. Right panel: Western blotting analysis was performed to measure the expression of Smad4 in HCC GR cells. **b** Left panel: Sequences of target sites for miR-130a-3p in Smad4 are shown. Right panel: Luciferase reporter assays were performed to identify the binding of miR-130a-3p to Smad4 3′-UTR. WT: wild type; Mut: mutation. *p < 0.05 vs control

To further determine the mechanism of miR-130a-3p-regulated invasion in HCC GR cells, we sought to identify the target of miR-130a-3p. According to the data from TargetScan, PicTar, and miRanda, Smad4 could be a potential target of miR-130a. Although it has been reported that miR-130a targeted Smad4 in granulocytic cells [43], another study did not support this report in human cancer cells [44]. Therefore, further investigation is required for validation of Smad4 as a miR-130a target. Our results from RT-PCR demonstrated that miR-130a-3p mimic treatment led to decreased Smad4 in HCC GR cells, whereas miR-130a-3p inhibitor treatment caused the up-regulation of Smad4 in HCC cells (Fig. 2a). Western blotting analysis further demonstrated that up-regulation of Smad4 was observed in HCC cells after miR-130a-3p inhibitor treatment (Fig. 2b). Consistently, the down-regulation of Smad4 was showed in HCC GR cells treated with miR-130a-3p mimic (Fig. 2b). In addition, we found high expression of Smad4 in HCC GR cells, which have lower expression of miR-130a-3p (Fig. 3a), suggesting that Smad4 could be a target of miR-130a-3p.

### Smad4 is a downstream target of miR-130a-3p

Bioinformatics analysis indicated that the Smad4 3′UTR harbors potential miR-130a-3p target sites (Fig. 3b). To further verify the Smad4 as a potential target of miR-130a-3p, we conducted the reporter assay in HCC cells with the luciferase gene driven by either wild-type or mutated Smad4 3′UTR sequences. We found that it has a significant reduction in luciferase activity with wild-type Smad4, but not mutant Smad4, in HepG2 GR cells transfected with miR-130a-3p mimic (Fig. 3b). Consistently, miR-130a-3p inhibitors treatment led to increased luciferase activity with wild-type Smad4 in HepG2 cells (Fig. 3b). Taken together, these findings further validate that Smad4 is a downstream target of miR-130a-3p.

### Down-regulation of Smad4 reverses EMT to MET in HCC GR cells

**Fig. 4:**
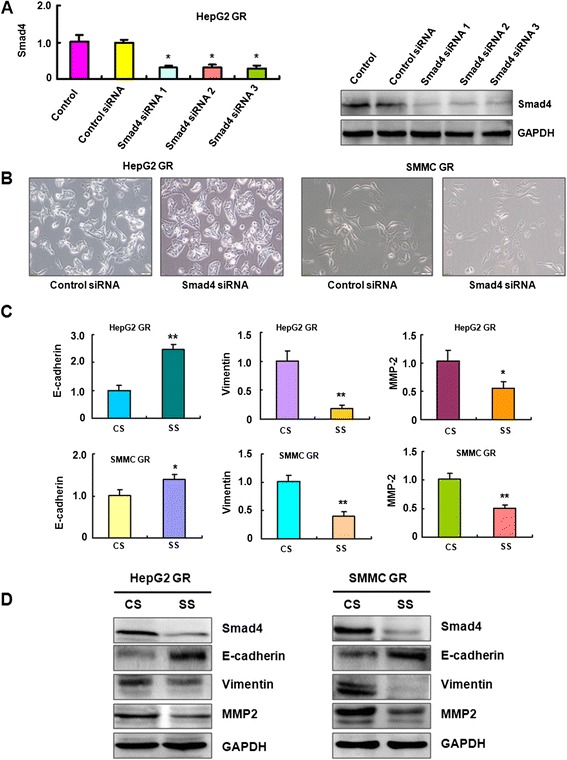
Down-regulation of Smad4 reverses EMT to MET in HCC GR cells. **a** Left panel: Real-time RT-PCR assay was used to detect the mRNA level of Smad4 in HepG2 GR cells transfected with Smad4 siRNAs. Right panel: Western blotting analysis was performed to measure the expression of Smad4 in HepG2 GR cells after Smad4 siRNA transfection. **b** Down-regulation of Smad4 caused reversal of EMT phenotype of HCC GR cells. HepG2 GR and SMMC-7721 GR cells transfected with control siRNA exhibited a fibroblastic-type phenotype, while these GR cells transfected with Smad4 siRNA display round-like epithelial cell shape. **c** HCC GR cells transfected with control siRNA or Smad4 siRNA were used for assessing the expression of EMT markers using Real-time RT-PCR. * *P* < 0.05, ** *P* < 0.01 vs control. **d** HCC GR cells transfected with control siRNA or Smad4 siRNA were used for assessing the expression of EMT markers by Western blotting analysis. CS: control siRNA; SS: Smad4 siRNA

Our previous study has shown that HCC GR cells have EMT phenotype [40]. To determine whether Smad4 plays a key role in GR-mediated EMT, we depleted the Smad4 using its specific siRNAs in HepG2 GR and SMMC 7721 GR cells. We found significant down-regulation of Smad4 by its siRNAs using RT-PCR and Western blotting analysis, respectively (Fig. 4a). Next, we used Smad4 siRNA1 to conduct the following experiments. We observed that both HepG2 GR and SMMC 7721 GR cells with Smad4 siRNA transfection displayed round cell-like morphology (Fig. 4b), suggesting that depletion of Smad4 reversed EMT to MET (mesenchymal to epithelial transition) in HCC GR cells.

### Depletion of Smad4 regulated EMT markers in HCC GR cells

To further confirm the reversal of EMT by knockdown of Smad4 in HCC GR cells, we detected the expression of EMT molecular markers. Our RT-PCR results showed that depletion of Smad4 decreased E-cadherin mRNA, but increased Vimentin and MMP2 mRNA levels in HepG2 GR and SMMC 7721 GR cells (Fig. 4c). Consistently, the results from our Western blotting validated that E-cadherin was increased, while Vimentin and MMP2 are down-regulated after depletion of Smad4 in HepG2 GR and SMMC 7721 GR cells (Fig. 4d). These results suggest that down-regulation of Smad4 caused the reversal of EMT to MET phenotype.

### Down-regulation of Smad4 inhibited cell migration and invasion in HCC GR cells

**Fig. 5:**
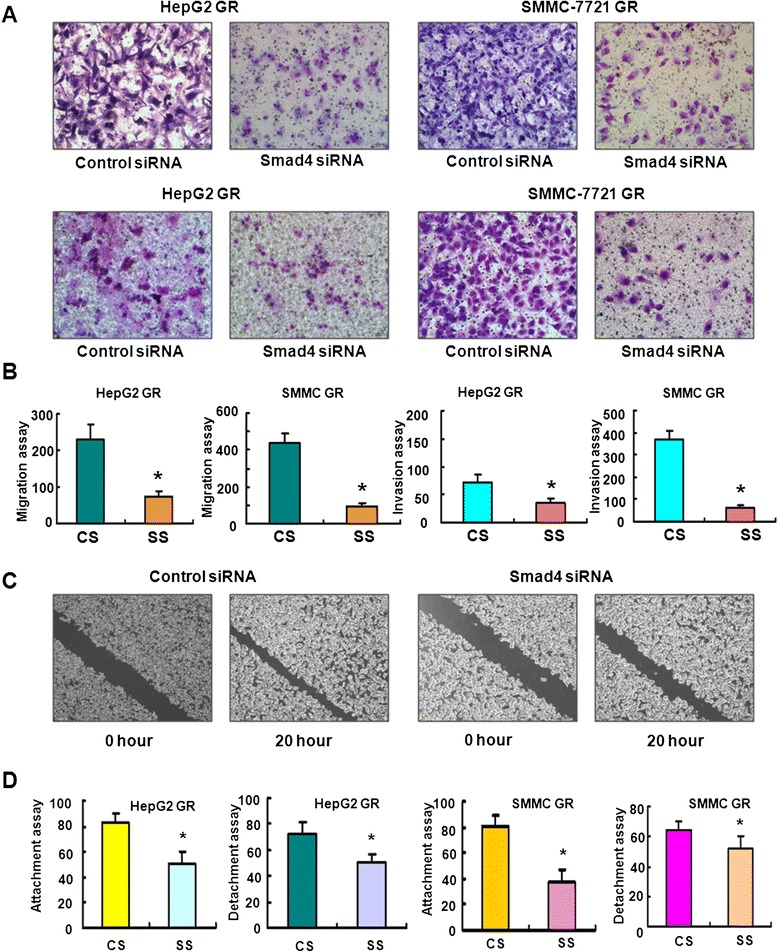
Down-regulation of Smad4 inhibited cell migration and invasion in HCC GR cells. **a** Top panel: Transfection of HepG2 GR cells and SMMC-7721 GR cells with Smad4 siRNA inhibited cell migration. Bottom panel: Smad4 siRNA suppressed cell invasion of HepG2 GR and SMMC-7721 GR cells by Transwell invasion assay. **b** Quantitative results are illustrated for panel **a. c** SMMC-7721 GR cells transfected with Smad4 siRNA caused decreased motility capacity as assessed by Wound healing assay. **d** HepG2 GR and SMMC-7721 GR cells transfected with Smad4 siRNA inhibited the attachment and detachment of cells. * *P* < 0.05 vs control siRNA

To explore whether Smad4 is involved in regulation of cell motility, we performed the migration and invasion assays in HCC GR cells after Smad4 siRNA transfection. We found that Smad4 siRNA transfecton led to inhibition of cell migration and invasion in both HepG2 GR and SMMC 7721 GR cells (Fig. 5a and b). The wound-healing assay further demonstrated that depletion of Smad4 suppressed cell motility in both HCC GR cells (Fig. 5c). In support of this, Smad4 siRNA treatment inhibited cell detachment and attachment in HepG2 GR and SMMC 7721 GR cells (Fig. 5d). Altogether, Smad4 plays a pivotal role in cell migration and invasion in HCC cells.

### Over-expression of miR-130a-3p enhances GR cells to gemcitabine sensitivity

**Fig. 6:**
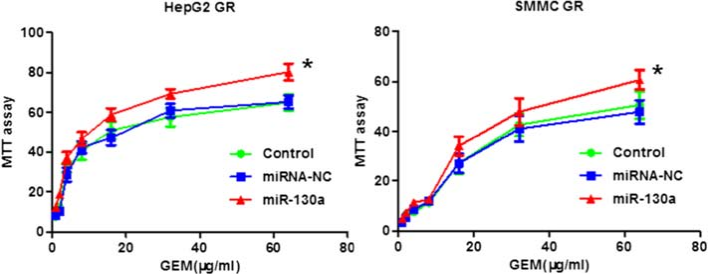
Over-expression of miR-130a-3p enhances GR cells to gemcitabine sensitivity. MTT assay was performed in GR cells treated with miR-130a-3p mimics. *, *P* < 0.05 compared with control and miRNA-NC

To investigate whether reintroduction of miR-130a-3p enhances GR cells to gemcitabine sensitivity, we performed MTT assay in miR-130a-3p mimics transfected GR cells. We found that reintroduction of miR-130a-3p in HepG2 GR cells significantly attenuated cell growth inhibition from 60 to 80 % induced by 65 μg/ml gemcitabine (Fig. 6). Similar result was observed in SMMC GR cells (Fig. 6). These results suggested that miR-130a-3p mimics-transfected GR cells were significantly more sensitive to gemcitabine-induced cell growth inhibition.

## Discussion

Recent studies have highlighted the important role of miR-130a in HCC progression and metastasis [45–47]. One study showed that miR-130a regulated the HBV-associated estrogen receptor alpha in human HCC cells [48]. miR-130a expression is correlated with pathological tumor grade in HCC [49]. Li et al. found that miR-130a expression was significantly down-regulated in HCC [45]. Moreover, lower expression of miR-130a was associated with overall survival of patients with HCC [45], suggesting that miR-130a could serve as a potential prognostic biomarker for HCC patients. Remarkably, it has been discovered that upregulated miR-130a increased drug resistance by targeting RUNX3 and Wnt signaling in cisplatin-treated HCC cells [47]. Our current study revealed that miR-130a-3p was down-regulated in HCC GR cells and overexpression of miR-130a-3p inhibited cell migration and invasion.

The data presented here demonstrated that miR-130a-3p-regulated EMT and cell invasion through inhibition of Smad4 in HCC GR cells. Recent studies have identified critical roles of Smad4 in tumorigenesis and EMT processes [50, 51]. Some studies have demonstrated that Smad4 plays tumor suppressive functions in most types of human cancers [52, 53]. However, multiple studies have indicated that Smad4 has oncogenic functions in several kinds of human cancers [54]. It is clear that Smad4 exerts a tumor-promoting role in HCC. Specifically, knockdown of Smad4 significantly reduced the colony formation and migratory capacity of HCC cells [54]. Moreover, overexpression of Smad4 was observed in HCC patient tumors compared with adjacent tissues [54]. Furthermore, knockdown of Smad4 significantly increased E-cadherin expression in prostate cancer cells [50]. Qiao et al. reported that miR-34a inhibited EMT in human cholangiocarcinoma by targeting Smad4 via TGF-beta/Smad signaling pathway [51]. In line with these findings, our results showed that knockdown of Smad4 inhibits cell migration, and invasion in HCC GR cells. Altogether, inactivation of Smad4 could be a novel strategy for the treatment of HCC.

A number of observations demonstrated that some small molecules and natural compounds could regulate the expression of miRNAs [55–57]. Multimodal synthetic small molecules have been developed to target the production of oncognic miRNAs [56]. Arsenic trioxide has been discovered to down-regulate miR-125b expression in huma glioma [57]. More importantly, natural compound curcumin has been reported to modulate miR-203-mediated regulation of the Src-Akt axis in bladder cancer [55]. Similarly, genistein inhibited cell growth and invasion via regulation of miR-27a and miR-34a in pancreatic cancer cells [58, 59]. Therefore, it is required to develop small molecules and discover compounds to control the expression of miRNAs in the near future. To this end, we found for the first time that miR-130a-3p was decreased in HCC GR cells. Moreover, we validated that miR-130a-3p targeted Smad4 expression. Notably, we confirmed that Smad4 plays a critical role in regulation of migration and invasion and EMT in HCC GR cells. Strikingly, reintroduction of miR-130a-3p enhances GR cells to gemcitabine sensitivity. However, transfection of mutant Smad4 without a miR-130a-3p binding site in HepG2 cells led to gemcitabine resistance (data not shown), suggesting that Smad4 plays a critical role in miR-130a-3p-mediated gemcitabine sensitivity. Altogether, our findings provide an insight into the molecular basis involved in gemcitabine resistance. However, further exploration is necessary to fully understand the molecular insights on miR-130a-mediated drug resistance and EMT in HCC cells.

## Conclusion

In summary, our study describes a potential mechanism for dysregulation of Smad4 by miR-130a-3p in HCC. Taken together, upregulation of miR-130a-3p and inactivation of Smad4 could be a promising approach for achieving better treatment of HCC.
